# Measures to Prevent and Control COVID-19 in Skilled Nursing Facilities: A Scoping Review

**DOI:** 10.1001/jamahealthforum.2024.5175

**Published:** 2025-01-31

**Authors:** Benjamin E. Canter, Agne Ulyte, Brian E. McGarry, Michael L. Barnett

**Affiliations:** 1 Department of Occupational Therapy, Sargent College of Health & Rehabilitation Sciences, Boston University, Boston, Massachusetts; 2 RAND Europe, Cambridge, United Kingdom; 3 Department of Medicine, University of Rochester, Rochester, New York; 4 Department of Health Policy and Management, Harvard T. H. Chan School of Public Health, Boston, Massachusetts; 5 Department of Medicine, Division of General Internal Medicine and Primary Care, Brigham and Women’s Hospital, Boston, Massachusetts

## Abstract

**Importance:**

Skilled nursing facilities (SNFs) experienced high mortality during the COVID-19 pandemic, leading them to adopt preventive measures to counteract viral spread. A critical appraisal of these measures is essential to support SNFs in managing future infectious disease outbreaks.

**Objective:**

To perform a scoping review of data and evidence on the use and effectiveness of preventive measures implemented from 2020 to 2024 to prevent COVID-19 infection in SNFs in the US.

**Evidence Review:**

Two analyses were performed. First, an analysis of the federal COVID-19 SNF Database was conducted to describe time trends in COVID-19 incidence, deaths, testing, vaccination, and treatment among SNF residents and staff, as well as shortages in staff and personal protective equipment (PPE). Then, a comprehensive literature search was conducted from May 2023 to April 2024 to identify high-quality evidence on the use and effectiveness of modifiable preventive measures used among SNF residents. Both nonpharmacologic (facility characteristics, PPE, cohorting, and isolation, visitation, staffing, testing) and pharmacologic (vaccination, treatment) measures were reviewed.

**Findings:**

Nationwide data indicated early shortages of PPE, infrequent testing, and persistent staff shortages in SNFs. Other nonpharmacologic measures, such as visitor restrictions or ventilation modifications, were widely adopted but there were no available national data to quantify their effectiveness. These nonpharmacologic measures lacked high-quality studies to verify effectiveness. In contrast, the effectiveness of vaccination and antiviral treatment was shown in multiple studies. Evidence also showed associations between COVID-19 outcomes in residents and crowding, staff size, hours per residents and networks, and surveillance testing of residents and staff. Despite high initial uptake, up-to-date vaccination status was suboptimal in residents and staff from 2022 to 2024. Only a minority of infected residents received antiviral treatment.

**Conclusions and Relevance:**

This scoping review found that although many preventive measures were implemented in SNFs in the US throughout the COVID-19 pandemic, few were based on clear evidence of their effectiveness. Pharmacologic measures, such as vaccination and antiviral treatment, had more robust evidence supporting their efficacy than nonpharmacologic interventions. Using the scarce resources and staff of SNFs on measures of questionable effectiveness could distract from known effective ones such as vaccination and antiviral treatment. When possible, implementation efforts should be commensurate with the demonstrated effectiveness of available preventive measures.

## Key Points

**Question:**

What is the evidence on preventive measures implemented to prevent COVID-19 in skilled nursing facilities?

**Findings:**

This scoping review identified 16 preventive measures, both nonpharmacologic (eg, staffing, visitor restrictions) and pharmacologic (eg, vaccines, antivirals) interventions. Nonpharmacologic measures were widely implemented but lacked evidence for effectiveness, whereas vaccinations and antivirals showed substantial benefits but were underutilized; up-to-date vaccination status was suboptimal in residents and staff and only a minority of infected residents received antiviral treatment.

**Meaning:**

These findings indicate that many preventive measures were implemented in skilled nursing facilities, but few had clear evidence of effectiveness; future implementation should be commensurate with demonstrated effectiveness.

## Teaser

This scoping review investigates the effectiveness of measures implemented in skilled nursing facilities in the US to prevent and control COVID-19 infections and deaths during the pandemic.

## Introduction

Nursing homes, or skilled nursing facilities (SNFs), are high-risk settings for COVID-19.^1^ In the US, the first outbreak in an SNF was reported on February 27, 2020, approximately 1 month after this country’s first-ever case of COVID-19 was reported.^2^ By the end of 2020, despite only representing 1% of the US population, SNF residents constituted 4% of all COVID-19 cases and 31% of its deaths.^3^ SNF residents are highly susceptible to COVID-19 infection because of their age, comorbidities, and frailty. The close-quarters environment of SNFs and the hands-on care required contributes to infection spread. Baseline staffing shortages and high turnover rates prior to the pandemic also made SNFs likely to experience COVID-19 outbreaks.^4^ These issues were compounded by expanding staff shortages, which were widely reported during the pandemic.^5^

The threat that COVID-19 posed to SNF residents led to rapid implementation of myriad nonpharmaceutic interventions. Although many of these measures (eg, PPE, cohorting, and isolation of infected individuals) were motivated by theoretical assumptions of effectiveness in SNFs, most had not specifically been tested in SNFs settings. For instance, SNFs restricted visitors, stopped communal activities (including meals in dining rooms), applied “cohorting” (grouping patients by infection status), isolated individual infected residents, adopted widespread surveillance testing, and required the use personal protective equipment (PPE) to limit infection spread. These actions were rooted in proven public health principles such as minimizing unneeded in-person contacts, yet SNFs face a number of unique challenges that make their effectiveness uncertain. For instance, SNF residents are dependent on staff and close physical contact to assist with mobility and personal care activities, so pure isolation practices are impossible. Furthermore, shortages of PPE, space, and staff caused further incomplete implementation of these general infection control efforts.^6^

As COVID-19 becomes endemic, we must evaluate best practices for respiratory disease mitigation in SNFs.^7^ Other infectious respiratory diseases (eg, influenza, respiratory syncytial virus, etc) remain leading causes of illness and death among residents and there is always a risk of future outbreaks from new viral threats. Yet, considerable uncertainty remains regarding evidence-based practices for preventing COVID-19 and other respiratory viruses in SNFs. Common narratives about low quality in SNFs are insufficient to explain how COVID-19 evolved in the sector.^8^

We conducted a scoping review paired with analysis of comprehensive national data. The purpose of this scoping review and data analysis was to summarize the literature on modifiable factors that could mitigate the impact of COVID-19 in SNFs and data on SNF efforts to address COVID-19 during the pandemic.

## Methods

This scoping review did not require institutional review board review because it was not human subjects research and used only publicly available data. The review protocol was not preregistered. We followed the PRISMA Extension for Scoping Reviews (PRISMA-ScR) reporting guidelines.

### Scope of the Review

We focused on modifiable measures, including nonpharmacologic interventions, antiviral treatments, and vaccination, that were implemented or recommended for COVID-19 prevention and outcome improvement in SNFs from 2020 to 2023. We identified regulations, recommendations,^9,10,11^ and reviews,^12,13,14,15,16^ and extracted modifiable measures at the resident or facility level. Selected measures were grouped into 3 groups—nonpharmacologic, pharmacologic, and miscellaneous system-level measures—and 16 subgroups (domains). Nonpharmacologic measures included resident- and facility-level interventions, such as PPE use, cohorting, isolation practices, and visitor restrictions; pharmacologic measures referred to the use of vaccines, antiviral treatments, and other medications aimed at reducing COVID-19 risk; miscellaneous system-level measures encompassed broader policies, such as state-level vaccination mandates, which affect nursing home operations at a regulatory or systemic level rather than through direct facility management.

### Data Analysis

We characterized the national context of COVID-19 in SNFs, including COVID-19 cases and deaths among residents, by collecting weekly data from the US Centers for Disease Control and Prevention (CDC) National Healthcare Safety Network (NHSN) database.^17^ This database contains data submitted by mandate on a weekly basis by US skilled nursing facilities to the CDC. We also used this database to characterize the prevalence of key modifiable measures to prevent COVID-19 in SNFs, such as PPE and staffing shortages over time, vaccination and testing among residents and staff, and antiviral treatment use among residents. Data analyses were performed from March 2023 to April 2024 using Stata, version 17.0 (StataCorp).

### Literature Search

We performed PubMed searches for nonpharmacologic, pharmacologic, and miscellaneous system-level interventions within SNFs for COVID-19. Title screening was done by 1 author (A.U. or B.E.C.), and supplemented by review with all authors of screening results to resolve uncertain cases. The search was conducted from May 2023 to April 2024. Detailed search strategy development, search terms, and selection criteria are provided in eTables 1 to 4 in Supplement 1.

### Evidence Selection and Synthesis

We reviewed results in a narrative scoping review format due to studies’ heterogeneity and the dearth of high-quality studies for most domains. Prevalence was evaluated directly for domains with available information in the NHSN database (staffing, PPE, testing, vaccination, and treatment). Domains not measured through NHSN (eg, facility characteristics, visitation, cohorting), were evaluated through a literature search for prevalence and effectiveness. We focused primarily on US studies with rigorous designs.^12^

### Literature Search Overview

We identified 3284 candidate titles, resulting in 188 full-text articles read. For a flow diagram of studies selected for inclusion, see eFigure 1 in Supplement 1.

## Results

We included 96 articles after the literature search. The Table presents a summary of the identified modifiable measures, their implementation prevalence, and evidence regarding their effectiveness.^6,18,19,20,21,22,23,24,25,26,27,28,29,30,31,32,33,34,35,36,37,38,39,40,41,42,43,44,45,46,47,48,49,50,51,52,53,54,55,56,57,58,59,60,61,62,63,64,65^

**Table.** Measures Associated With COVID-19 Outcomes in Skilled Nursing Facilities (SNFs)

Domain and subdomains	Modifiable measure	SNF status during COVID-19 pandemic	Association with COVID-19 outcomes
**Nonpharmacological measures**			
Facility, physical, and social environment	Crowding	Most rooms in SNFs were shared or semiprivate.^18^	More residents per room, smaller living area per bed, and higher occupancy rate were associated with more COVID-19 cases and worse outcomes among residents and staff in some^19,20,21^ but not all studies.^22,23,24^
	Ventilation	Many SNFs lacked adequate ventilation and filtration systems.^18^	A single noncontrolled study of COVID-19 cases and deaths in 81 SNFs found no statistically significant decrease in cases after installation of UV air purification systems.^25^
PPE	PPE supply	PPE was lacking in 1 in 5 facilities in early July 2020,^6^ persisted up to 2021. From June 7, 2020, to March 7, 2021, 20% of 15 148 SNFs admitted residents despite experiencing notable PPE shortages.^26^	PPE supply not associated with staff cases, except for surgical mask supply^21^ and N95 masks.^27^
Cohorting and isolation practices	Cohorting of infected residents	Wide range from 9% (Colorado)^18^ to 78% (Michigan),^28^ 94% (sample of SNFs from 19 states),^29^ and 97% (Texas)^30^ in 2020. Some facilities with dedicated space did not cohort all infected patients.^31^	Nonadherence to quarantine guidelines on admission of new residents did not result in more COVID-19 outbreaks.^32^
	Dedicated staff for patients with COVID-19	Wide range of facilities with fully separated staff for patients with COVID-19, from 3% (Colorado)^18^ to 91% (sample of SNFs from 19 states).^29^	SNFs with staff caring for both patients infected and not infected with COVID-19 had higher risk of COVID-19 outbreaks (not adjusting for community incidence).^33^
Visitor policies	Visitor restrictions	Visitor restrictions were universally implemented across SNFs in early 2020.^29,34^	A study of 11 858 nursing homes found that statewide visitation bans were not significantly associated with staff COVID-19 cases.^21^
Staffing	Staff availability per resident-day	One in 5 facilities reported staffing shortages in early July 2020,^6^ and beyond, into 2023. Facilities were more likely to report staffing shortages up to 16 weeks after a COVID-19 outbreak.^35^	Most studies point to higher staffing per resident being associated with better outcomes (eg, fewer resident COVID-19 cases or deaths).^20,36^ Better staffing was associated with better COVID-19 outcomes in already infected patients or fewer total cases—but not the probability of at least 1 COVID-19 case in the SNF.^22,24,27,37^
	Staff size per SNF	Wide variation in staff size, even conditional on bed size.^38^	Staff size (No. of unique clinical and other workers in a facility on a given day) was associated with higher COVID-19 cases.^38^
	Staffing networks across SNFs	Nationally, SNFs shared staff with an average of 7.1 other facilities in March-May 2020 in US.^39^	Shared staff associated with increased COVID-19 cases.^39^ Wards in facilities with staff employed in multiple facilities were more likely to have outbreaks in 2021, but not 2020.^33^
Testing	Testing and screening of staff and residents	Most facilities performed less than 1 test per staff member per week in 2020-2022.	Higher testing facilities had fewer resident cases and deaths, especially before vaccine availability.^40^ Mandated employee testing was associated with a decrease of cases within 3 wk in a single state study.^41^
**Pharmacological measures**			
Vaccination	Resident vaccination	As of December 10, 2023, 33% of SNF residents had up-to-date vaccination against COVID-19, compared to 72% and 10% of residents being vaccinated for influenza and respiratory syncytial virus, respectively.^42^	Hosting a vaccination clinic in a facility earlier led to faster decrease in COVID-19 cases.^43,44,45^ Although the effectiveness against infection waned over time,^46^ it remained high against severe disease and mortality.^47^ The third vaccine dose (first booster) and bivalent booster were effective against infection and severe disease. ^48,49,50,51,52^
	Staff vaccination	In July 2021, 60% of staff were fully vaccinated; certified nursing aides had a 49% vaccination rate.^53^	Between and within facility variation in staff vaccination rates were associated with significant reductions in resident cases and deaths. ^54,55^
Treatment	Antiviral COVID-19 treatment	From June 2021-December 2022, only 18% of diagnosed residents received oral antiviral or monoclonal antibody treatment. By the end of 2022, this proportion increased only to 25%.^56^	Studies on treatment outcomes in residents were limited to a preventive trial of a monoclonal antibody^57^ and a few small retrospective studies.^58,59^ A single large retrospective cohort study reported large reduction in hospitalizations and severe disease outcomes among patients with COVID-19 using either molnupiravir or nirmatrelvir/ritonavir.^60^
**Miscellaneous measures**			
System level	Unions	Health care worker unions were present in 17% of SNFs in 2020-2021.^61^	Facilities with health care worker unions in New York in 2020 had lower COVID-19 mortality and fewer reported PPE shortages.^62^ Nationally, smaller mortality decreases were found.^61^
	Private-equity ownership	In 2020, 4.7% of SNFs were private-equity owned.^63^	PPE shortages were 10%-30% more likely in private-equity owned facilities compared to others, but there were no significant differences in COVID-19 cases or deaths in early 2020.^63^
	Green House model	Approximately 300 Green House homes were serving 3200 residents.^64^	In first half of 2020, Green House and comparable small SNFs had lower COVID-19 infection and fatality rates than the nearest traditional facilities.^65^

Abbreviation: PPE, personal protective equipment.

### Nonpharmacologic Measures

Nursing facilities used various nonpharmacologic measures to reduce the risk of SARS-CoV-2 introduction and spread. Nonpharmacologic measures were the only means of disease mitigation in early 2020, before vaccination and treatments.

#### Staffing

**Figure 1.**
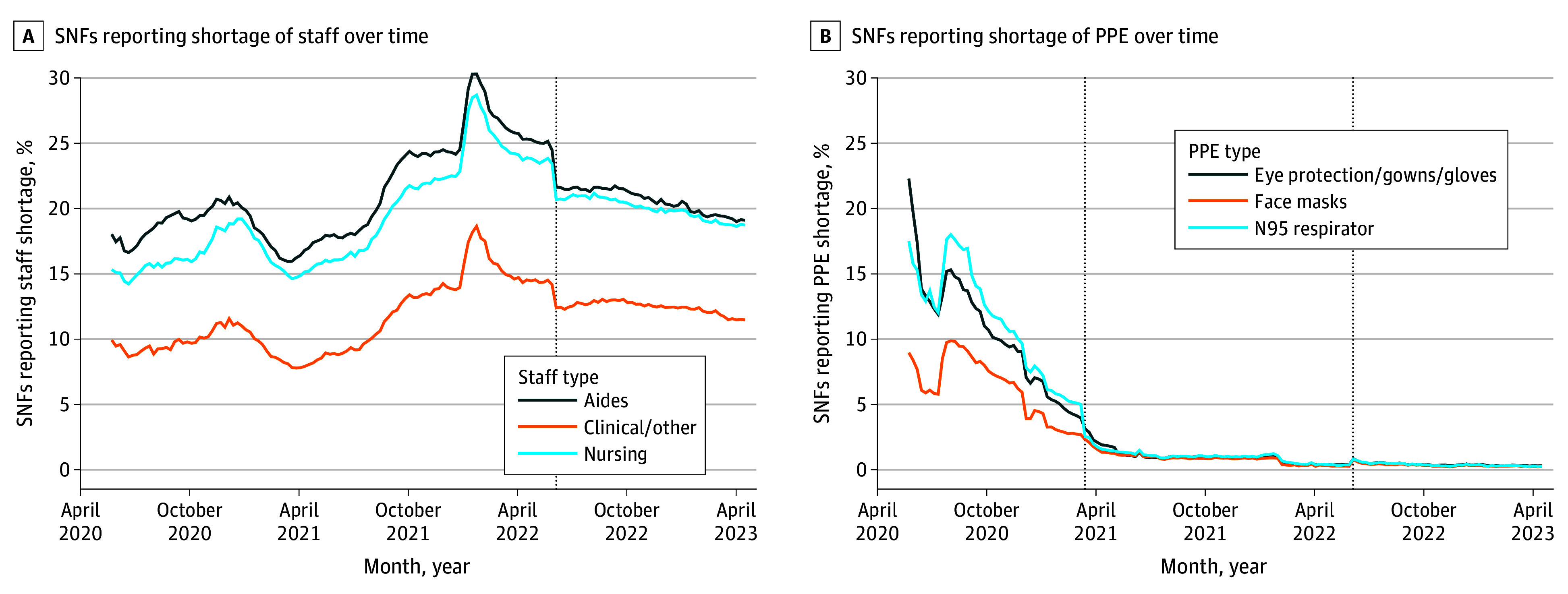
Skilled Nursing Facilities (SNFs) Reporting Shortage of Personal Protective Equipment (PPE) and Staff Over Time Fluctuations in staffing shortages (A), with notable peaks in early 2022, followed by a general decline in shortages for aides and clinical/other staff, and a more stable trend for nursing staff significant decline in reported shortages for all types of PPE over the observed period. (B) Shortages of staff equipment and N95 respirators over 12 months. The vertical dotted line in both panels marks the point when the phrasing of the question in the survey or the recording of the response in the dataset was changed.

Staffing shortages, contributing to lower-quality care years before the pandemic, worsened in 2020. Staffing decreases were augmented during COVID-19 outbreaks by temporary absences and permanent departures.^28^ Figure 1A shows that approximately one-fifth of facilities reported staffing shortages in July 2020.^6^ Shortages increased through 2022, the latest data available. The size of the SNF workforce has not recovered to prepandemic levels, although staff to resident ratios appear to have returned to prepandemic levels.

Multiple studies evaluated the associations between staffing and COVID-19 outcomes (Table). Most found that higher staffing, measured as hours per day, were associated with fewer resident cases and deaths,^22,36,37,66^ consistent with more staff facilitating increased infection control protocol adherence (eg, PPE, etc). The association between staffing and COVID-19 mitigation appears to be multidimensional. The number of individuals working in a facility was a stronger predictor of infection risk than direct care hours.^38^ Facilities with more staff per resident were more likely to experience outbreaks. Similarly, staff working across multiple facilities (or units within 1 facility) is predictive of higher risk of infection spread.^2,39^

Although facility staffing levels may not be an easily modifiable measure because of labor market shortages, COVID-19 *strike force* staffing teams were deployed as a temporary measure to improve staffing. Additionally, infection control improvement could be obtained through other more easily modifiable dimensions such as shifting workers to full-time schedules and limiting staff from working in more than 1 facility.

#### Personal Protective Equipment

Widespread personal protective equipment (PPE) use was recommended in health care settings throughout the pandemic, but 20% of facilities experienced PPE shortages into 2021, likely preventing increased PPE use (Figure 1B).^6^ However, many facilities with PPE shortages continued to admit residents. Evidence of PPE effectiveness on COVID-19 prevention in SNFs was largely limited to cross-sectional studies, with inconsistent results. Several studies reported staff nonadherence to PPE use protocols.^29,34,67,68^ A cross-sectional study of 13 156 SNFs found a significant association between N95 mask shortages and prevalence of any COVID-19 case, but no US studies have evaluated adherence and COVID-19 incidence.^27^ Furthermore, no US studies have compared the importance of different PPE types (eg, gloves, masks, eyewear, or gowns).^29,34,67,68^ A study conducted in France including 2076 cases of health care workers and matched controls found that eyewear and gowns were protective against COVID-19; apron use was associated with slight increases in staff infection incidence.^69^ These findings are detailed in the Table.

#### Testing

**Figure 2.**
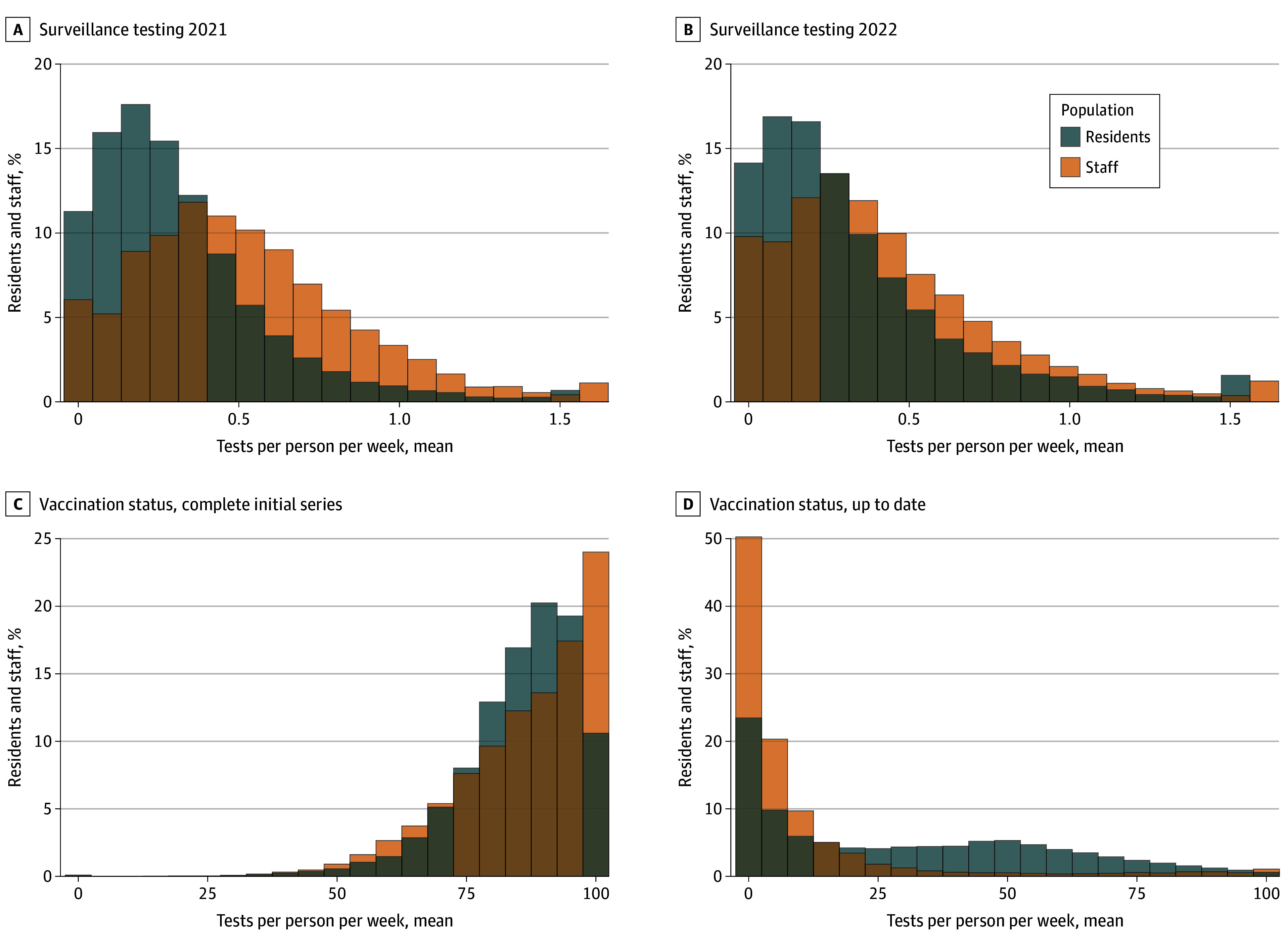
Distribution of Testing and Vaccination of Residents and Staff Across Skilled Nursing Facilities (SNFs) Mean number of COVID-19 surveillance tests per person per week in SNFs for staff and residents in 2021 and 2022 shows changes in testing frequency over 2 years (A and B). Vaccination status of staff and residents showing the completed initial vaccination series and the percentage of individuals who are up to date, highlighting the distribution of vaccination coverage between the 2 populations (C and D).

Testing residents and staff in SNF serves 2 purposes: to preventively identify asymptomatic cases and accurately diagnose symptomatic persons. Due to supply shortages, regular testing was limited in 2020; many facilities restricted testing to symptomatic cases or only residents,^28,70^ testing solely for diagnosis, not surveillance. Test result turnaround times, often 3 days or longer in 2020, likely reduced preventive testing’s efficacy. Variation of testing intensity persisted in 2021 to 2022 with the majority of SNFs performing fewer than 1 screening test per week for staff or residents (Figure 2A).

The association between increased testing and increased identified COVID-19 cases makes it difficult to directly assess the effect of testing on case prevention. One study used facilities’ staff testing volume in weeks without new COVID-19 cases (relative to neighboring facilities without outbreaks) as a proxy for testing propensity, to capture testing variations not attributable to new cases or time-varying changes in recommendations or availability.^40^ Prior to vaccine availability, facilities with the highest test volumes had 12% and 14% fewer COVID-19 cases and deaths per outbreak, respectively, than SNFs with the lowest testing volumes. The number of deaths per outbreak did not differ based on whether SNFs predominantly used laboratory-based polymerase chain reaction (PCR) or antigen-based tests. In facilities using PCR, faster turnaround time was associated with fewer resident deaths.

Another study compared the number of COVID-19 cases in Florida SNFs, before and after a semimonthly testing mandate was implemented in June 2020.^41^ A 10% increase in testing frequency was associated with a 1% decrease in COVID-19 cases. However, without a control group of nonmandated facilities, causal assumptions are limited.

#### Visitor Policies and Social Restrictions

Visitor restrictions were widely implemented across SNFs, resulting in a 34% decrease in overall foot traffic to facilities.^71^ Evidence for visitor restrictions’ effectiveness was scarce, as national implementation prevented a comparison group. Many SNFs aimed to facilitate virtual visits to substitute the loss of in-person contacts to maintain residents’ quality of life,^28^ yet only 22% of persons living in assisted living or SNFs reported weekly contact with family or friends, a decrease from 56% in 2019.^72^ Furthermore, restrictions limited access to health care workers deemed nonessential (eg, hospice practitioners).^73^ No studies assessed the effectiveness of restricted social activities (eg, communal dining, group activities) in SNFs, despite their widespread implementation during the early pandemic.

#### Cohorting and Isolation Practices

Physically separating patients with COVID-19 from other residents either individually (isolation) or in groups (cohorting) was not implemented universally, due to physical space constraints and staffing shortages (Table). Multiple studies reported that adherence to quarantine guidelines on admissions did not result in fewer outbreaks after adjusting for community incidence of COVID-19.^32^ A cross-sectional study of 9081 SNFs in England found that dedicating specific staff solely to patients with COVID-19 was associated with reduced COVID-19 outbreak risk.^33^

#### Physical Facility Environment and Crowding

The typical size and layout of US SNFs and resident rooms leads to multiple contacts with other residents and staff. Crowding, measured as the average number of residents per room and bathroom in a SNF, higher bed occupancy, or facilities with shared bedrooms, was linked to higher COVID-19 prevalence in most studies (Table). Importantly, while the probability of COVID-19 introduction did not differ between facilities based on crowding levels, increased crowding predicted the number of COVID-19 cases and deaths among facilities with preexisting outbreaks.^19^

Poor air filtration and ventilation may amplify the risks of close living quarters. However, evidence on the association of air management technologies in SNFs, beyond a single noncontrolled study of 81 SNFs,^25^ is lacking. Suboptimal hand hygiene was reported in nearly a third of SNFs in 2020,^29^ but no research addressed its association with outcomes. Lack of controlled studies limits our ability to draw conclusions regarding the impact of crowding and the physical environment on infection spread.

### Pharmacologic Measures

**Figure 3.**
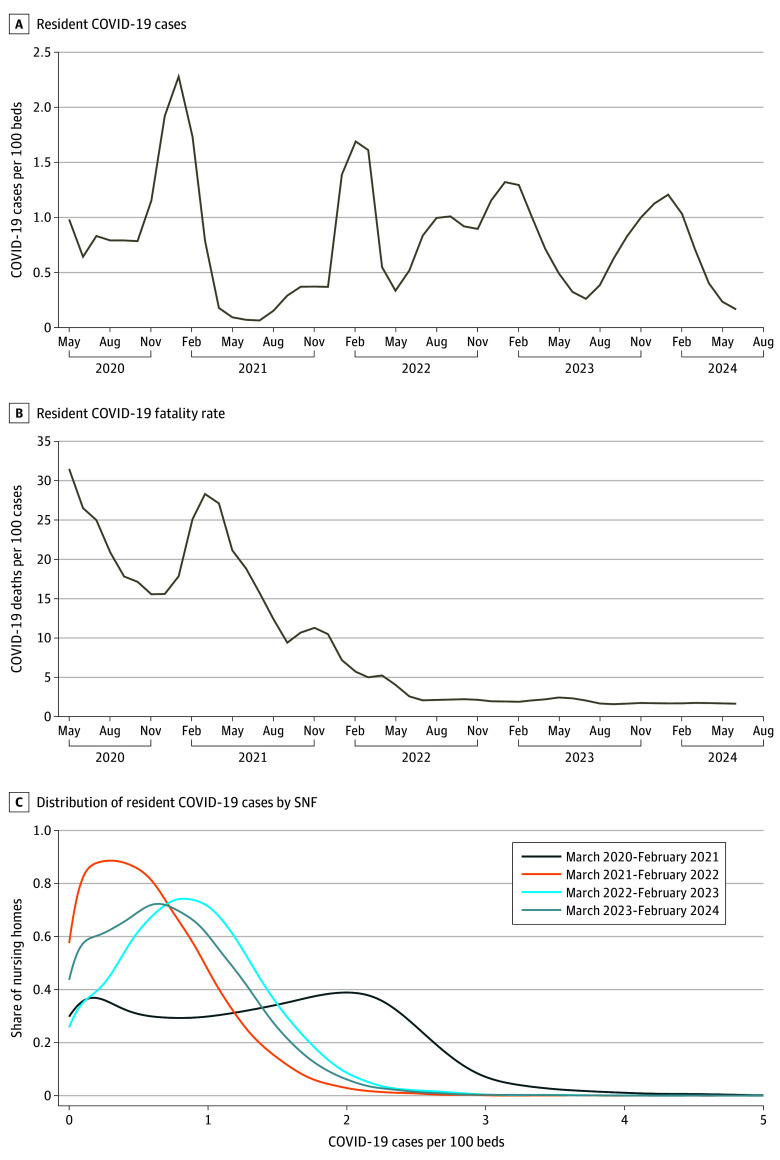
COVID-19 Cases and Mortality Among Skilled Nursing Facilities (SNFs) Residents From 2020 to 2023 Monthly national incidence of COVID-19 cases in all SNF residents, per every 100 beds, from May 2020 to May 2024, demonstrates a seasonal fluctuation in rates (A). Mortality rate of all SNF residents from May 2020 to May 2024 (B), with rates spiking in early 2021 and steadily declining from April 2021 through May 2022. Distribution of COVID-19 cases per 100 beds in SNFs (C) across 4 time periods: March 2020-February 2021, March 2021-February 2022, March 2022-February 2023, and March 2023-February 2024. Each curve represents the share of SNFs experiencing different levels of COVID-19 case rates, highlighting shifts in the distribution of cases and improvements in controlling outbreaks over time.

Eventually, the spread of COVID-19 and related mortality fell after the majority of residents were vaccinated in early 2021 (Figure 3). Pharmacologic measures—vaccination and antiviral treatment—were pivotal in reducing spread. High-quality randomized clinical trials (RCTs) demonstrated high potential to improve COVID-19 outcomes in the SNF population.

#### Vaccination of Residents

Vaccination of SNF residents started in December 2020. By July 2021, 81% were fully vaccinated, but the rate of take-up substantially varied (Figure 2B). This variation grew as additional rounds of vaccination became recommended and the CDC started tracking up-to-date vaccination status. Clinical trials in the community population demonstrated that initial vaccination reduced COVID-19 cases and mortality.^74,75^ Although no RCTs evaluated SNF resident vaccination and COVID-19−related mortality, retrospective cohort studies found similar results in the SNF population as the trials in community-dwelling adults (Table).

Waning vaccine-induced immunity and increasing natural immunity reduced the effectiveness of the primary vaccination series over time,^46,47^ but booster vaccinations remained highly effective at preventing severe outcomes and deaths.^48,49,50,51,52,76^ However, the length of booster efficacy remains an area of controversy.^52,76^ A study found it may be shorter than the original vaccination series.^76^ Effectiveness over time may vary by vaccine type; 1 retrospective cohort study of 4315 residents found that while the RNA-1273 and BNT162b2 vaccinations demonstrated similar efficacy within 150 days of vaccination, RNA-1273 reduced risk of resident infection by 44% more than BNT162b2 after 150 days.^77^

#### Vaccination of Staff and Vaccination Mandates

Staff vaccination prevalence was lower than resident vaccination rates, particularly beyond the primary series (Figure 2B), despite its ability to reduce staff infections by up to 83.4%.^78^ Higher staff vaccination coverage was associated with 56% and 19% reductions in risk of COVID-19 infections and deaths in residents per 10% of staff vaccinated (Table). A longitudinal cohort study of 15 042 SNFs found that increases of 10% in staff vaccination reduced resident and staff cases and deaths prior to the Omicron variant in December 2021, but was not associated with improved outcomes during the Omicron period.^54^ Although several studies examined strategies to increase staff vaccination uptake, no effective intervention was identified. Although survey and nonrandomized studies found educational campaigns were associated with increased vaccination coverage,^79,80,81^ a cluster randomized trial using similar educational techniques did not significantly improve staff or resident vaccination rates.^82^

Many SNFs implemented staff vaccination mandates early in 2021. By summer 2021, several states enacted mandates, followed by a federal mandate in early 2022. While many feared mandates would cause staff resignations, both state^83,84,85^ and federal^86^ mandates increased staff vaccination rates by several percentage points, without notable impact on staffing levels. Vaccination coverage remained suboptimal even after mandates were enacted.^83,84^

#### Antiviral and Antibody Treatment

We identified no RCTs on antiviral and antibody treatments in SNF residents. However, 2 RCTs performed in community-dwelling populations demonstrated these treatments may have strong potential to improve the outcomes of COVID-19.^87,88^ Meanwhile, a large retrospective cohort study in Hong Kong^89^ found molnupiravir or nirmatrelvir/ritonavir use was associated with reduced hospitalizations, intensive mechanical ventilation, and death in SNF residents (Table). However, treatment was underutilized in SNFs in the US. Although monoclonal antibodies reduced the risk of hospitalization and death in early strains of COVID-19,^58^ only an estimated 30% of infected residents received this treatment by 2021, when theoretically close to 100% of COVID-19 cases could have benefitted from treatment.^89^ When easier-to-administer oral antiviral treatments (ie, nirmatrelvir/ritonavir and molnupiravir) became available in 2022, use rates remained low. By March 2023, only approximately 35% of resident COVID-19 cases were treated with antivirals (eFigure 2 in Supplement 1).

## Discussion

This scoping review identified 16 modifiable measures that could potentially reduce the burden of COVID-19 in SNFs and found that most lacked direct evidence on effectiveness. Some of the most widespread and restrictive interventions, such as visitor restrictions, isolation of residents, and the suspension of communal activities, were implemented in SNFs despite the lack of studies specifically assessing their effectiveness in reducing COVID-19 transmission in these settings. Meanwhile, evidence was robust for pharmacologic measures, including staff and resident vaccination and antiviral treatments, but they were underused in SNFs. In particular, oral antiviral COVID-19 treatments were underused despite the ease of treatment administration, wide availability, and subsidized costs before late 2023.^90,91^ The underuse of vaccination and treatment and widespread use of visitor bans suggest that implementation efforts were not tied to measures’ demonstrated effectiveness.

The potential contributors for the difference in evidence between pharmacologic and nonpharmacologic interventions are variable, but likely reflect greater financial and research backing for pharmacologic studies, which often require rigorous testing. Meanwhile, nonpharmacologic measures, like visitor restrictions, were straightforward for facilities to implement but were difficult to test with quasi-experimental or experimental techniques due to the nearly universal use of these measures and ethical considerations.

Despite substantial efforts to implement COVID-19 mitigation measures, few were systematically or nationally tracked. Almost none used randomized or staggered roll-out methods to facilitate causal inference. While understandable given the frantic response to the pandemic in 2020, the lack of attention to intervention assessment early on reflects a missed opportunity to identify best practices for respiratory illness risk mitigation. One exception was the creation of the SNF COVID-19 module within the NHSN survey, without which we would know considerably less about the pandemic’s impact on SNFs.

Another critical issue is that staff were a critical vector through which COVID-19 entered SNFs but faced many challenges. First, staff had even lower vaccination rates than residents despite vaccine availability and no associated costs.^92^ Staffing shortages, high turnover, and staff working across multiple institutions compounded staff vaccination challenges, likely exacerbating infection rates. Second, higher staffing ratios were associated with better clinical outcomes; reduced staff size was associated with fewer close contacts for infection spread. Despite COVID-19 “strike teams” of temporary staff and recent CMS changes to minimum staffing levels,^93^ consistent staffing patterns were likely just as important as total staffing levels. Increasing full-time facility-employed staff and reducing the use of part-time workers can reduce risk. Greater investment in staff and policies rewarding retention are needed to achieve staffing improvements that reduce risk of infection spread while maintaining care quality.^94^

Complete in-person visitor bans isolated residents from their families; socialization and autonomy were sacrificed for infection risk mitigation, despite risks of prolonged loneliness and violations in resident rights.^95,96^ Yet, the efficacy of the visitor bans remains unestablished. Nursing facilities should weigh the likely impact on residents’ quality of life, and strongly voiced resident and family preferences against autonomy-limiting interventions with unclear benefit. Given the evidence supporting surveillance testing of staff for preventing COVID-19 spread, rapid testing programs could be similarly applied to visitors and caregivers in future pandemics, balancing risk mitigation with maintaining resident quality of life.

Physical space and environment is also frequently discussed as a key factor in infection control. In this review, most evidence on SNF facility layout in COVID-19 cases came from international studies. Smaller facilities and private rooms by design result in smaller resident and staff cohorts and fewer unique contacts per resident without reductions in time of direct care. Residents also prefer these designs as they increase dignity, autonomy, and function.^97^ However, infrastructure changes require major monetary and political investment.

### Limitations

Study bias was not assessed with a standardized tool. The focus on US studies limits international generalizability, but this scoping review is the first to our knowledge to successfully assess the prevalence and efficacy of COVID-19 mitigation and treatment strategies in US SNFs.

## Conclusions

While various preventive measures were implemented in SNFs throughout the COVID-19 pandemic, there was little direct evidence on effectiveness of most of these in the SNF setting. Pharmacologic measures such as vaccination and antiviral treatment had more robust evidence supporting their efficacy than nonpharmacologic interventions. Using the scarce SNF resources and staff to implement measures of questionable effectiveness could distract from effective interventions such as vaccination. When possible, future infection control implementation efforts should focus on strategies with demonstrated effectiveness preventive measures. A multipronged, evidence-informed approach using environmental strategies, testing, early antiviral therapy, and vaccination is likely the most effective way to prevent cases and fatalities from COVID-19 and other respiratory infections in SNF residents. Systemic reform is needed to increase resource allocation toward SNFs to support changes known to mitigate respiratory infection risk and improve resident outcomes.

## Notes

Supplement 1. eMethods 1. Literature Search Strategy and Domains of COVID-19 Preventive Measures In Nursing Homes eTable 1. Domains of COVID-19 preventive measures in nursing homes eMethods 2. Search Terms and Article Screening eTable 2. Common and domain-specific search terms eMethods 3. Article Inclusion and Exclusion Criteria eTable 3. Exclusion criteria eTable 4. Number of screened titles, abstracts, and full text manuscripts by preventive measure domain eFigure 1. Inclusion and Exclusion Process of Screened Manuscripts eFigure 2. Proportion of SNF residents with COVID-19 diagnosis receiving COVID-specific medication treatment eReferences

Supplement 2. Data Sharing Statement
