# Supplementary material for: Measures to Prevent and Control COVID-19 in Skilled Nursing Facilities: A Scoping Review
Source: JAMA Health Forum. 2025 Jan 31;6(1):e245175. doi: 10.1001/jamahealthforum.2024.5175 (PMC11786235; doi:10.1001/jamahealthforum.2024.5175)
Supplement: Supplement 1. — eMethods 1. Literature Search Strategy and Domains of COVID-19 Preventive Measures In Nursing Homes eTable 1. Domains of COVID-19 preventive measures in nursing homes eMethods 2. Search Terms and Article Screening eTable 2. Common and domain-specific search terms eMethods 3. Article Inclusion and Exclusion Criteria eTable 3. Exclusion criteria eTable 4. Number of screened titles, abstracts, and full text manuscripts by preventive measure domain eFigure 1. Inclusion and Exclusion Process of Screened Manuscripts eFigure 2. Proportion of SNF residents with COVID-19 diagnosis receiving COVID-specific medication treatment eReferences [file jamahealthforum-e245175-s001.pdf]

Supplemental Online Content

Canter BE, Ulyte A, McGarry BE, Barnett ML. Measures to Prevent and Control COVID-19 in Skilled Nursing Facilities. *JAMA Health Forum.* Published online January 31, 2025. doi:10.1001/jamahealthforum.2024.5175

eMethods 1. Literature Search Strategy and Domains of COVID-19 Preventive Measures In Nursing Homes

eTable 1. Domains of COVID-19 preventive measures in nursing homes

eMethods 2. Search Terms and Article Screening

eTable 2. Common and domain-specific search terms

eMethods 3. Article Inclusion and Exclusion Criteria

eTable 3. Exclusion criteria

eTable 4. Number of screened titles, abstracts, and full text manuscripts by preventive measure domain

eFigure 1. Inclusion and Exclusion Process of Screened Manuscripts

eFigure 2. Proportion of SNF residents with COVID-19 diagnosis receiving COVID-specific medication treatment

eReferences

This supplemental material has been provided by the authors to give readers additional information about their work.

© 2025 Canter BE et al. *JAMA Health Forum*

Literature search strategy

The purpose of the literature search was to identify studies describing COVID-19 preventive measures in nursing homes in 2020-2024 and to capture the frequency of their implementation (in the US) and/or their effectiveness at preventing infection and severe outcomes (US and other countries). The search was structured under eight domains, described in the table below.

eTable 1 Domains of COVID-19 preventive measures in nursing homes

		**Relevant research questions on:**	
**Domain**	**Preventive measures or circumstances**	**Prevalence of measures**	**Outcomes of measures**
Facility characteristics	Features of nursing homes that potentially prevented or contributed to COVID-19 infection spread: hand hygiene, surface cleaning, measures to reduce crowding in common spaces, measures for dining and group activities, air management.	How often the different hand, surface, air decontamination strategies were applied?<br>How often group activities and dining were restricted?	How the implementation of such measures contributed to decrease in COVID-19 infections in residents or staff?
Cohorting and isolating practices	Isolating COVID-19 positive residents in single rooms; if not available – cohorting several infected residents together.<br>Keeping cohorts of residents and staff separate wthin the nursing home.	How often and what type of cohorting of confirmed and suspected COVID-19 cases was used in nursing homes for COVID-19 prevention?	What were the preventive effects of isolating and cohorting COVID-19 cases?
Personal protective equipment (PPE)	Using masks and other PPE for nursing home staff, residents, visitors.	How often were PPE available in nursing homes?<br>How often were they used properly?	Did supply and use of PPE reduce risk of COVID-19 incidence and spread within nursing homes?
Visitor policies	Restricting visitors, volunteers to enter nursing homes.	How widely was the restriction of visiting and volunteering implemented?	Did restricting visitors and volunteers entering nursing homes

			reduce COVID-19 incidence among residents?
Staffing	Staff size, unique staff count, staff training, staff cohorting, infection control specialist activities.	What is the typical staff size and unique count in nursing homes?<br>How widespread are staffing shortages and other staffing factors related to COVID-19 spread?<br>How often there is infection control specialist in a nursing home?	What characteristics of staff in nursing homes were related to higher risk of COVID-19 infections and worse outcomes for residents and staff?
Testing	Acute infection (viral) testing among residents, staff, visitors.	How often was screening and diagnostic testing done in nursing home residents and staff?	What was the preventive effect of screening testing on COVID-19 prevalence?<br>How testing influenced resident outcomes?
Vaccination	Vaccination of residents and staff.	Which percentage of residents and personnel were vaccinated over time in nursing homes?<br>Which measures were effective at increasing vaccination rates?	What were the clinical outcomes (prevented infections and severe outcomes) of residents, depending on the personal, other residents', and staff vaccination?
Treatment	Use of effective antiviral treatment for COVID-19 infected nursing home residents.	What was the prevalence of treatment use among COVID-19 infected residents?	What was the effect on COVI-19 infected resident outcomes of using antiviral treatment?

While a few other domains were identified in the recommendations and reviews (e.g., data collection and reporting requirements or communication with the local health department), they were considered too indirect for evaluation of their effectiveness to prevent COVID-19 case or improve health outcomes, and thus excluded from this review.

© 2025 Canter BE et al. *JAMA Health Forum*

© 2025 Canter BE et al. *JAMA Health Forum*

Search Terms and Article Screening

Systematic literature search was done in PubMed database. A separate search was run for each domain of measures. Keywords were identified through a discussion among authors, based on relevant pre-identified publications and considering the inputs of a group of experts (Joseph G. Allen, DSc, MPH, CIH, Vincent Mor, PhD, MEd, Tamara Konetzka, PhD, Rachel M. Werner, MD, PhD, David C. Grabowski, PhD, and Karl E. Steinberg, MD). Table below presents the search terms. Each search combined the terms to capture COVID-19, nursing home context, and domain specific terms using AND operators. All terms were searched in Title or Abstract.

eTable 2 Common and domain-specific search terms

**Terms common to all searches**	**Search terms**
Capturing COVID-19	COVID OR SARS-CoV-2 OR coronavirus
Capturing nursing homes context	"nursing home*" OR "skilled nursing" OR SNF OR "long term care facilit*" OR (("community living center*") AND (veteran*)) OR "care home*"
**Terms specific to preventive measures domains**	
Facility characteristics	"hand hygiene" OR "hand washing" OR surface* OR disinfection OR "physical barrier*" OR crowding OR overcrowding OR communal OR dining OR "group activit*" OR air OR ventilat* OR airflow OR filtration OR pressur*
Cohorting and isolating practices	(cohorting OR transfer* OR isolat* OR occupancy OR confinement OR congregate OR "single room")
Personal protective equipment (PPE)	PPE OR "personal protective equipment" OR mask* OR glove* OR gown* OR "face shield*" OR N95 OR respirator OR respirators OR "fit testing"
Visitor policies	visiting OR visitation OR visitor* OR volunteer* OR family
Staffing	staffing OR staff OR "nursing hours" OR "care hours" OR "nurse hours" OR "infection control specialist*" OR "infection control and prevention" OR "registered nurse*" OR "licensed practical nurse" OR RN OR LPN OR "certified nurse aide*" OR "nursing assistant*" OR CNA or "staff size" or "staff shortage*" or "staff network*" or "personnel" or "employee*"
Testing	test* OR "point of care" OR surveillance OR screening OR turnaround OR antigen OR "polymerase chain reaction" OR PCR

© 2025 Canter BE et al. *JAMA Health Forum*

Vaccination	vaccine OR vaccination OR immunization OR immunisation OR Janssen OR "J&J" OR "Ad26.COV*" OR Pfizer OR BioNTech OR comirnaty OR Moderna OR spikevax OR Novavax OR "NVX-CoV2373*" OR Nuvaxovid OR Covovax OR mandat*
Treatment	nirmatrelvir OR ritonavir OR paxlovid OR remdesivir OR veklury OR molnupiravir OR lagevrio OR bamlanivimab OR etesevimab OR casirivimab OR imdevimab OR sotrovimab OR bebtelovimab
Prophylaxis	prophylaxis OR Evusheld OR Tixagevimab OR cilgavimab

We limited the search to studies published in 2020 or later, and those in English. In this review, we focused on the US and countries that could inform its policy, and while the restriction on language means that we might have missed some studies outside the US, it was pragmatic given the scoping and rapid nature of the review. We also used a filter to exclude reviews.

Searches were run from May 11, 2023 to April 20, 2024, and titles were screened by a single author (Ulyte, Canter) to select abstracts for more detailed screening. Title screening was done to exclude studies that are very unlikely to have relevance, erring to keep potential false positive entries. Table below summarizes the exclusion criteria. If at least one exclusion criterion was identified, the study was excluded from further analysis.

© 2025 Canter BE et al. *JAMA Health Forum*

Article Inclusion and Exclusion Criteria

eTable 3 Exclusion criteria

**Category**	**Details**
Nursing home setting or residents	Research does not concern nursing homes (for domains of vaccination, testing, facility, staff and PPE) or nursing home residents or staff (treatment, visiting, cohorting).<br>We exclude studies set in schools, prison systems, camps, as well as studies focusing only on assisted living facilities and communities, inpatient rehabilitation facilities, and pediatric populations.
Case reports and series	A single reported facility ("a nursing home") or less than 10 nursing homes included.
Study or publication type	Reviews, preprints, communications other than full papers (e.g., conference abstracts), trial protocols, and qualitative research studies were excluded. We also excluded studies if no abstract was available on PubMed (e.g., commentary pieces).
Exposure or preventive measure	Research does not concern with the effect of the exposures or the preventive measures of interest (depending on the domain; e.g., does not concern staffing in the domain on staff).
COVID-19	Study not related to the topic of COVID-19 prevention or outcomes such as incidence, morbidity, or mortality.<br>This criterion was used primarily to exclude studies without a clear preventive measure and focusing purely biochemical markers (e.g., antibody titers), except if they were used to capture COVID-19 diagnosis; and studies on vaccine adverse effects, without reference to COVID-19 outcomes.<br>We also excluded studies capturing outcomes only before the COVID-19 pandemic (e.g., in 2019 and earlier) or only the period in 2020 before vaccination was available (for vaccination domain).

In cases of uncertainty, the title was included to clarify the scope further. Abstracts were screened by a single author (Ulyte, Canter) on May 19 and April 20, 2024, using the same exclusion criteria. Duplicates were eliminated at the stage of manuscript screening. A table showing the number of screened titles, abstracts, and full text manuscripts is provided below.

We identified 3284 candidate titles, resulting in the screening of 825 abstracts and 188 full text manuscripts.

© 2025 Canter BE et al. *JAMA Health Forum*

eTable 4 Number of screened titles, abstracts, and full text manuscripts by preventive measure domain

	Number of screened		
**Domain**	**Titles**	**Abstracts**	**Manuscripts**
Facility characteristics	191	44	12
Cohorting and isolating practices	387	66	19
Personal protective equipment (PPE)	170	67	17
Visitor policies	298	49	8
Staffing	829	179	41
Testing	885	177	17
Vaccination	488	118	58
Treatment	27	11	6
Prophylaxis	9	0	0

Note: titles and abstracts contained duplicates between the domains. However, they were considered separately for each domain where they were identified. Duplicates were eliminated at the manuscript stage, so that the number of manuscripts included in this table (and screened for inclusion) is the count of unique manuscripts. As no abstracts were identified for prophylactic pharmacological treatment, the domain was not included in the manuscript separately.

Further 19 titles were identified via manual and reference search, with 10 abstracts and manuscripts included for further analysis. A total of 188 manuscripts were read in detail to verify their inclusion (based on the same exclusion criteria as for titles and abstracts) and extract key information on study methods and results. A single author (Ulyte, Canter) extracted the country and region of the study, the number of nursing homes, residents and staff studied, study design, and analytical methods. We noted if the study reported only the prevalence of the measure, its effects, or both. We categorized the studies according to their design and analytical methods into 1) experimental (e.g., randomized trials), 2) pseudo-experimental (e.g., studying the effects of natural variation in exposure), 3) observational studies using regression models adjusting for geographically and temporally specific community incidence of COVID-19, 4) observational studies using regression models without such adjustments, 5) person-level studies (relevant only to pharmacological preventive measures: vaccination and treatment), and 6) other.

After information extraction, we excluded studies with design likely leading to biased results (e.g., small convenience samples of nursing homes; studies without a control group or comparator), studying nursing home residents or staff only among a larger population and not including their sub-group results, and studies without clear description of their methods.

The remaining studies were considered for relevance to each domain. Many of the studies (particularly, relying on observational data and regression models) were included in multiple domains. We aimed to reference all studies included after full manuscript consideration in the

review; however, we focused on high quality studies and did not include lower quality studies (e.g., reporting regression models not adjusted for community incidence of COVID-19) if more impactful evidence was available.

© 2025 Canter BE et al. *JAMA Health Forum*

eFigure 1 Inclusion and Exclusion Process of Screened Manuscripts

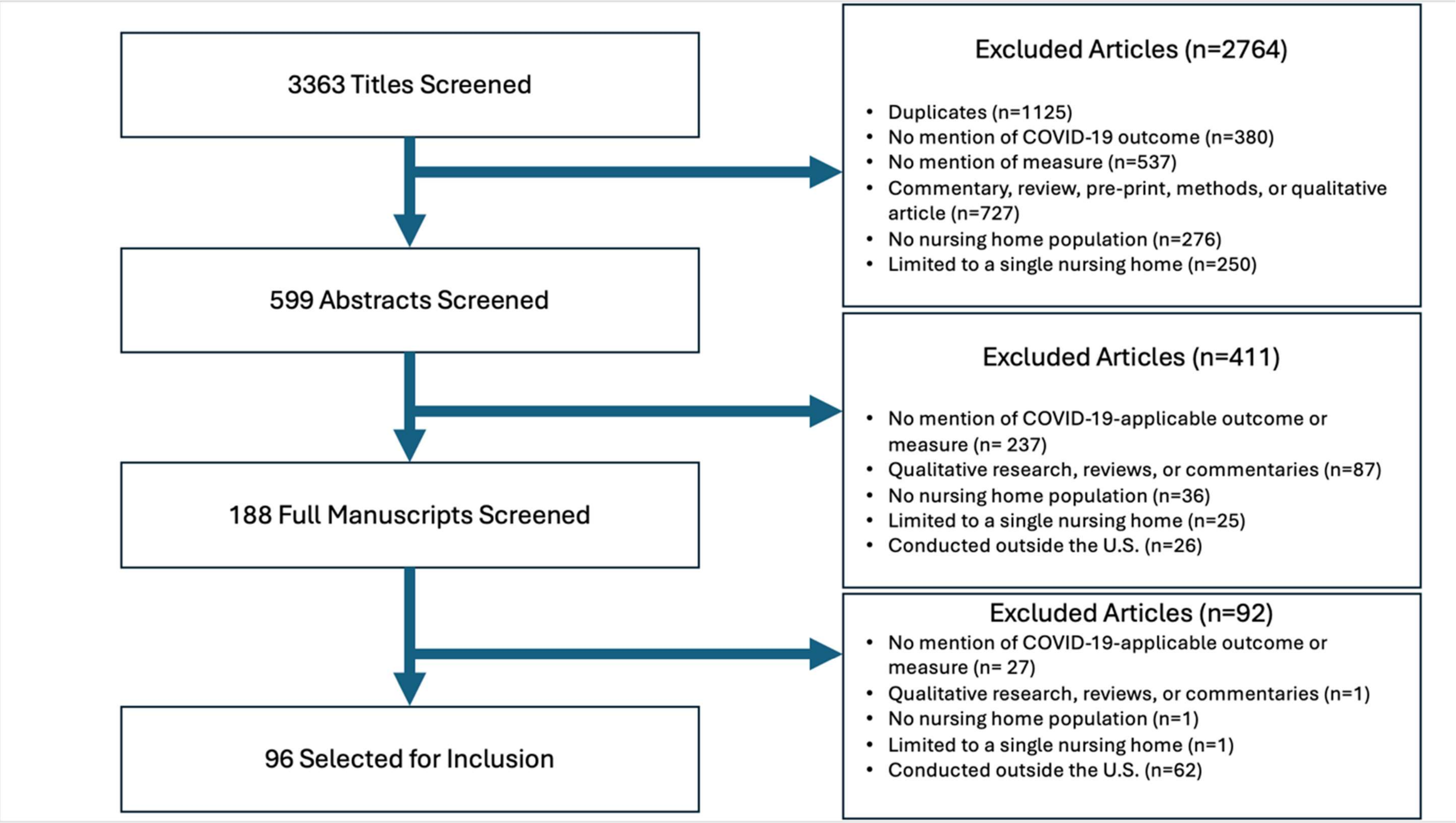

© 2025 Canter BE et al. *JAMA Health Forum*

eResults

eFigure 2 Proportion of SNF residents with COVID-19 diagnosis receiving COVID-specific medication treatment

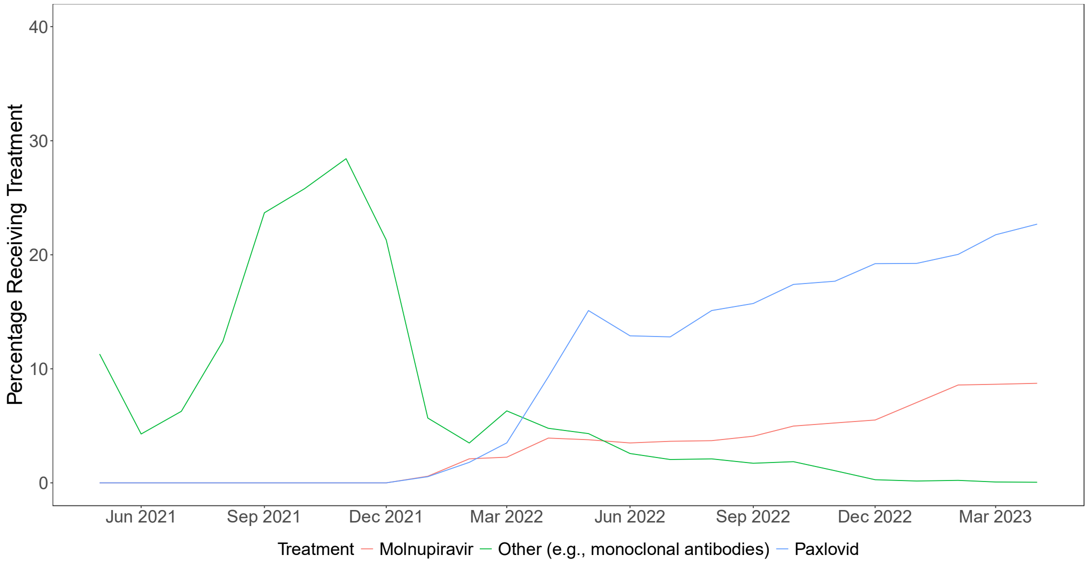

This graph shows the percentage of skilled nursing facility residents receiving different COVID-19 treatments from June 2021 to March 2023. The treatments are categorized into three types: Molnupiravir (red line), other treatments such as monoclonal antibodies (green line), and Paxlovid (blue line). The graph illustrates the trends in the use of these treatments over time, highlighting peak and decline in the use of monoclonal antibodies, the gradual increase in Molnupiravir and the rapid uptake in the use of Paxlovid starting in early 2022. Each treatment group was capped at 200% of overall resident COVID-19 cases in that month.

© 2025 Canter BE et al. *JAMA Health Forum*

eReferences

eReferences. Additional studies not cited in the main Article

- Chen MM, Grabowski DC. Intended and unintended consequences of minimum staffing standards for nursing homes. *Health Econ*. 2015;24(7):822-839. doi:10.1002/hec.3063
- Cantor J, Whaley C, Simon K, Nguyen T. US Health Care Workforce Changes During the First and Second Years of the COVID-19 Pandemic. *JAMA Health Forum*. 2022;3(2):e215217-e215217. doi:10.1001/JAMAHEALTHFORUM.2021.5217
- McGarry BE, Steelfisher GK, Grabowski DC, Barnett ML. COVID-19 Test Result Turnaround Time for Residents and Staff in US Nursing Homes. *JAMA Intern Med*. 2021;181(4):556-559. doi:10.1001/JAMAINTERNMED.2020.7330
- Lombardo FL, Bacigalupo I, Salvi E, et al. The Italian national survey on Coronavirus disease 2019 epidemic spread in nursing homes. *Int J Geriatr Psychiatry*. 2021;36(6):873-882. doi:10.1002/GPS.5487
- Shallcross L, Burke D, Abbott O, et al. Factors associated with SARS-CoV-2 infection and outbreaks in long-term care facilities in England: a national cross-sectional survey. *Lancet Healthy Longev*. 2021;2(3):e129-e142. doi:10.1016/S2666-7568(20)30065-9
- Torres ML, Díaz DP, Oliver-Parra A, et al. Inequities in the incidence and mortality due to COVID-19 in nursing homes in Barcelona by characteristics of the nursing homes. *PloS One*. 2022;17(6). doi:10.1371/JOURNAL.PONE.0269639
- Duval D, Palmer JC, Tudge I, et al. Long distance airborne transmission of SARS-CoV-2: rapid systematic review. *BMJ*. 2022;377. doi:10.1136/BMJ-2021-068743
- Antivirals, Including Antibody Products | COVID-19 Treatment Guidelines. Accessed January 14, 2024. https://www.covid19treatmentguidelines.nih.gov/therapies/antivirals-including-antibody-products/
- Benin AL, Soe MM, Edwards JR, et al. Ecological Analysis of the Decline in Incidence Rates of COVID-19 Among Nursing Home Residents Associated with Vaccination, United States, December 2020-January 2021. *J Am Med Dir Assoc*. 2021;22(10):2009-2015. doi:10.1016/j.jamda.2021.08.004
- Domi M, Leitson M, Gifford D, Nicolaou A, Sreenivas K, Bishnoi C. The BNT162b2 vaccine is associated with lower new COVID-19 cases in nursing home residents and staff. *J Am Geriatr Soc*. 2021;69(8):2079-2089. doi:10.1111/jgs.17224
- Mor V, Gutman R, Yang X, et al. Short-term impact of nursing home SARS-CoV-2 vaccinations on new infections, hospitalizations, and deaths. *J Am Geriatr Soc*. 2021;69(8):2063-2069. doi:10.1111/jgs.17176
- McGarry BE, Barnett ML, Grabowski DC, Gandhi AD. Nursing Home Staff Vaccination and Covid-19 Outcomes. *N Engl J Med*. 2022;386(4):397-398. doi:10.1056/NEJMc2115674
- McGarry BE, Sommers BD, Wilcock AD, Grabowski DC, Barnett ML. Monoclonal antibody and oral antiviral treatment of SARS-CoV-2 infection in US nursing homes. *JAMA*. 2023;330(6):561-563
- Rachel Nania. Are COVID Treatments Still Free? How to cover drug costs now that Uncle Sam is no longer footing the bill for everyone. AARP. January 8, 2024. Accessed June 9, 2024. https://www.aarp.org/health/drugs-supplements/info-2023/are-paxlovid-covid-treatments-still-free.html
- Centers for Medicare & Medicaid Services (CMS). Your Rights and Protections as a Nursing Home Resident. https://downloads.cms.gov/medicare/Your_Resident_Rights_and_Protections_section.pdf

- Bergman C, Stall NM, Haimowitz D, et al. Recommendations for Welcoming Back Nursing Home Visitors During the COVID-19 Pandemic: Results of a Delphi Panel. *J Am Med Dir Assoc*. 2020;21(12):1759-1766. doi:10.1016/J.JAMDA.2020.09.036
- Wu HH, Chien LJ, Su CH, Tseng SH, Chang SC. COVID-19 outbreaks in long-term care facilities-a nationwide population-based cohort study in Taiwan, May-July 2021. *J Formos Med Assoc Taiwan Yi Zhi*. 2023;122(12):1331-1337. doi:10.1016/j.jfma.2023.06.008
- Evers J, Geraedts M. Potential determinants of the quantity and duration of COVID-19 outbreaks in geriatric long-term care facilities. *BMC Geriatr*. 2023;23(1):759. doi:10.1186/s12877-023-04446-4
- Krutikov M, Stirrup O, Fuller C, et al. Built Environment and SARS-CoV-2 Transmission in Long-Term Care Facilities: Cross-Sectional Survey and Data Linkage. *J Am Med Dir Assoc*. 2024;25(2):304-313.e11. doi:10.1016/j.jamda.2023.10.027

© 2025 Canter BE et al. *JAMA Health Forum*
