# Supplementary material for: Measures to Prevent and Control COVID-19 in Skilled Nursing Facilities: A Scoping Review
Source: JAMA Health Forum. 2025 Jan 31;6(1):e245175. doi: 10.1001/jamahealthforum.2024.5175 (PMC11786235; doi:10.1001/jamahealthforum.2024.5175)
Supplement: Supplement 2. — Data Sharing Statement [file jamahealthforum-e245175-s002.pdf]

Data Sharing Statement

Canter. Measures to Prevent and Control COVID-19 in Skilled Nursing Facilities. *JAMA Health Forum*. Published January 31, 2025. doi:10.1001/jamahealthforum.2024.5175

Data
Data available: No

Additional Information
Explanation for why data not available: Not applicable
